# Factors Associated With Hyperuricemia in Patients With Coronary Heart Disease

**DOI:** 10.31083/RCM49042

**Published:** 2026-06-09

**Authors:** Qinyu Sun, Yifan Deng, Zhiyuan Gao, Shenghu He, Wei Zhou, Jing Zhang

**Affiliations:** ^1^Department of Cardiology, Northern Jiangsu People’s Hospital Affiliated to Yangzhou University, 225001 Yangzhou, Jiangsu, China; ^2^Department of Cardiology, Northern Jiangsu People’s Hospital, 225001 Yangzhou, Jiangsu, China; ^3^Department of Urology, Northern Jiangsu People’s Hospital Affiliated to Yangzhou University, 225001 Yangzhou, Jiangsu, China; ^4^Department of Rheumatology and Immunology, Affiliated Hospital of Yangzhou University, 225000 Yangzhou, Jiangsu, China

**Keywords:** coronary heart disease, hyperuricemia, correlation analysis, predictive model

## Abstract

**Background:**

This study aimed to investigate clinical factors associated with hyperuricemia (HUA) in patients with newly diagnosed coronary heart disease (CHD) and to evaluate the correlation of novel derived biomarkers with HUA, thereby providing a reference for clinical risk identification, prevention, and treatment.

**Methods:**

This retrospective study analyzed 2265 patients with newly diagnosed CHD (2019–2024) who were randomly assigned to a modeling group (n = 1812) and a validation group (n = 453); HUA was defined as serum uric acid (UA) ≥420 μmol/L. Least absolute shrinkage and selection operator (LASSO) regression and multivariate logistic regression were used to identify associated factors. A prediction model was constructed and evaluated using receiver operating characteristic (ROC) curves, decision curve analysis (DCA), and calibration plots.

**Results:**

Independent factors associated with HUA included triglycerides (odds ratio (OR) = 1.097, 95% CI: 1.036–1.160), creatinine (OR = 1.030, 95% CI: 1.025–1.035), monocyte-to-high-density lipoprotein cholesterol ratio (MHR; OR = 1.447, 95% CI: 1.057–1.981), remnant cholesterol (RC; OR = 1.812, 95% CI: 1.406–2.337), and alcohol use (OR = 1.596, 95% CI: 1.202–2.119) (all *p *< 0.05). The model demonstrated good discrimination with areas under the ROC curve (AUROCs) of 0.781 (0.729–0.832) in the modeling group and 0.780 (0.751–0.808) in the validation group, as well as good calibration and clinical utility at threshold probabilities of 0.22–1.00 and 0.20–0.66, respectively. MHR and RC exhibited a positive nonlinear relationship with HUA risk, with a stronger association for MHR in the hypertensive subgroup (*p *< 0.05).

**Conclusion:**

MHR and RC are novel biomarkers associated with HUA risk in patients with new-onset CHD. When combined with traditional factors such as triglycerides and creatinine, these novel biomarkers support a multifactorial, integrated evaluation of HUA-related risk and serve as a reference for clinical risk stratification and management.

## 1. Introduction

In recent years, coronary heart disease (CHD) has emerged as a major cardiovascular disease threatening human health. Its pathological features primarily include the deposition of lipids, calcium, and other substances within the coronary arterial walls, leading to the formation of atherosclerotic plaques. As these plaques progressively enlarge, they may cause narrowing or even occlusion of the coronary artery lumen [1]. Once the blood supply function of the coronary artery is compromised or lost, the myocardium may suffer injury or necrosis due to ischemia and hypoxia.

Hyperuricemia (HUA), a common metabolic disorder, often results from acquired factors such as high-purine diets, fructose intake, alcohol consumption, and myeloproliferative disorders, which lead to excessive uric acid (UA) production [2]. HUA results from an imbalance in UA production and elimination, constituting a classic metabolic disorder that impacts multiple organs and overall systemic metabolism [3]. Research indicates that HUA is strongly linked to a range of cardiovascular conditions, such as hypertension, atrial fibrillation, chronic kidney disease, heart failure, metabolic syndrome, and coronary artery disease [4]. At the molecular level, HUA drives the onset and progression of CHD via multiple pathways, including inflammatory activation, oxidative stress, insulin resistance, endoplasmic reticulum stress, and endothelial dysfunction [5]. Additionally, HUA can induce microvascular injury by stimulating the renin-angiotensin system (RAS), suppressing endothelial nitric oxide synthesis, and promoting vascular smooth muscle proliferation.

Currently, the factors associated with HUA and their relationship with emerging biomarkers remain unclear in patients with newly diagnosed CHD. Therefore, this study aims to systematically evaluate HUA-related risk factors and potential novel biomarkers in this patient population, with the goal of providing insights for clinical prevention and management.

## 2. Methods and Participants

### 2.1 Study Population and Grouping

This retrospective study enrolled patients diagnosed with CHD at Northern Jiangsu People’s Hospital between January 1, 2019, and July 31, 2024. All included patients were symptomatic (excluding those with acute coronary syndrome) and underwent elective coronary angiography, which revealed epicardial coronary artery diameter stenosis ≥50%. Subject selection was conducted based on predefined inclusion/exclusion criteria and data integrity verification.

**Fig. 1.**
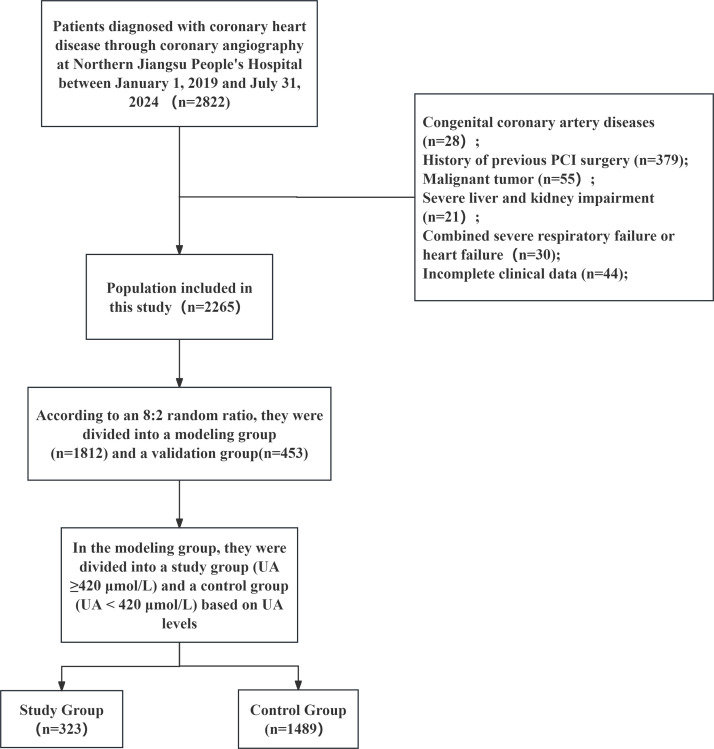
**Participant enrollment flowchart**. PCI, percutaneous coronary intervention; UA, uric acid.

Eligible subjects were randomly divided into a modeling group (n = 1812) and a validation group (n = 453) in an 8:2 ratio. Within the modeling group, patients were further classified into a study group (UA ≥420 μmol/L, n = 323) and a control group (UA <420 μmol/L, n = 1489), as illustrated in Fig. 1.

The study protocol was approved by the Ethics Committee of Northern Jiangsu People’s Hospital (No. 2025ky310).

Inclusion Criteria:

1. Age ≥18 years;

2. Undergoing coronary angiography for the first time;

3. Complete clinical data available;

4. No history of hereditary or familial diseases.

Exclusion Criteria:

1. Previous diagnosis of HUA or use of urate-lowering medications;

2. Severe hepatic impairment, defined as aspartate aminotransferase (AST) or alanine aminotransferase (ALT) levels ≥3 times the upper limit of normal, as well as chronic kidney disease (CKD) stages 4–5 [glomerular filtration rate (GFR) <30 mL/min].

3. Previous diagnosis of CHD, or prior percutaneous coronary intervention or coronary artery bypass grafting; New York Heart Association (NYHA) functional class III–IV heart failure, or cardiogenic shock;

4. Hyperthyroidism, severe infection, malignant tumor, immune system disorders, or hematologic diseases.

### 2.2 Clinical Data Collection and Calculation of Related Indicators

General patient information—including age, smoking history, alcohol use history, type 2 diabetes mellitus (T2DM), and hypertension—was collected from the electronic medical record system.

Hypertension was defined as systolic blood pressure ≥140 mm Hg and/or diastolic blood pressure ≥90 mm Hg measured on three separate occasions.

The diagnosis of T2DM was established based on any of the following criteria: a documented history of diabetes, current use of hypoglycemic agents, a fasting plasma glucose level of ≥7.0 mmol/L, or a 2-hour postprandial glucose level of ≥11.1 mmol/L.

Smoking was defined as consuming more than one cigarette per day for over six months.

Heavy alcohol use was defined as intake exceeding 50 mL per occasion, more than three times per week, for at least six consecutive months.

Venous blood samples were collected promptly after admission to measure routine hematologic parameters such as hemoglobin and white blood cell count. Following a 12-hour fast, additional venous blood was drawn the next morning to determine fasting glucose and lipid profiles.

The following novel inflammatory biomarkers were calculated:

PLR: platelet-to-lymphocyte; LMR: lymphocyte-to-monocyte; NLR: neutrophil-to-lymphocyte; PAR: platelet-to-albumin; PHR: platelet-to-HDL cholesterol; MHR: monocyte-to-HDL cholesterol; CHR: high-sensitivity C-reactive protein (hs-CRP)-to-HDL cholesterol; SII: systemic immune-inflammation index (platelets × neutrophils/lymphocytes); SIRI: systemic inflammation response index (monocytes × neutrophils/lymphocytes); AISI: aggregate index of systemic inflammation (neutrophils × monocytes × platelets/lymphocytes); CALLY: CRP-albumin-lymphocyte index (albumin × lymphocytes / hs-CRP); IBI: inflammatory burden index (hs-CRP × neutrophils).

Metabolism-related biomarkers included: TyG index: ln(triglycerides × fasting blood glucose) / 2; TyG-BMI: TyG index × body mass index; AIP: atherogenic index of plasma [log(triglycerides / HDL-C)]; Non-HDL-C: total cholesterol − HDL-C; NHHR: non-HDL-C/HDL-C ratio; RC: remnant cholesterol (total cholesterol − HDL-C − LDL-C); PNI: prognostic nutritional index [serum albumin (g/L) + 5 × total lymphocyte count (×10^9^/L)].

### 2.3 Statistical Analysis

All statistical analyses were conducted using R 4.1.1 (R Foundation for Statistical Computing, Vienna, Austria). Continuous variables with normal distribution are expressed as mean ± standard deviation and compared using the independent samples *t*-test, while non-normally distributed variables are presented as median (interquartile range) and analyzed with the Mann-Whitney U test. Categorical variables are reported as frequency (percentage) and assessed by the chi-square test.

To identify factors independently associated with HUA in new-onset CHD patients, least absolute shrinkage and selection operator (LASSO) regression was first used for variable selection, followed by multivariate logistic regression. Multicollinearity among novel biomarkers was evaluated by variance inflation factor (VIF), with only variables exhibiting VIF <5 retained in the final model to ensure reliability.

The nomogram’s predictive performance was evaluated based on discrimination, calibration, and clinical utility: discrimination was measured by the area under the curve(AUC), calibration was tested using the Hosmer-Lemeshow test and calibration plots, and clinical utility was assessed via decision curve analysis (DCA). *p* < 0.05 indicated statistical significance.

## 3. Results

### 3.1 Comparison of Baseline Characteristics in the Modeling Group

The modeling group included a total of 1812 participants, comprising 1489 individuals in the control group and 323 in the study group. Comparison of baseline characteristics between the two groups revealed that the following parameters were significantly higher in the study group than in the control group: proportion of males, history of hypertension, history of alcohol consumption, White Blood Cell count (WBC), Monocyte count (MON), Triglycerides (TG), Apolipoprotein B (Apo B), Creatinine (Cr), and CRP. In contrast, Apolipoprotein A1 (Apo A1) and High-density lipoprotein-Cholesterol (HDL-C) levels were significantly lower in the study group compared to the control group. No statistically significant differences were observed in the remaining baseline indicators. Detailed results are presented in Table 1.

**Table 1.** **Comparison of baseline characteristics between the two groups**.

Variables		Total (n = 1812)	Control group (n = 1489)	Study group (n = 323)	Statistic	*p*
Gender, n (%)					χ^2^ = 21.861	<0.001
	Female	461 (25.442)	412 (27.670)	49 (15.170)		
	Male	1351 (74.558)	1077 (72.330)	274 (84.830)		
Hypertension, n (%)		1242 (68.543)	996 (66.891)	246 (76.161)	χ^2^ = 10.579	0.001
Atrial fibrillation, n (%)		258 (14.238)	214 (14.372)	44 (13.622)	χ^2^ = 0.122	0.727
Diabetes, n (%)		456 (25.166)	378 (25.386)	78 (24.149)	χ^2^ = 0.216	0.642
Smoking, n (%)		974 (53.753)	785 (52.720)	189 (58.514)	χ^2^ = 3.584	0.058
Drinking, n (%)		468 (25.828)	363 (24.379)	105 (32.508)	χ^2^ = 9.155	0.002
Age [M (IQR), year]		65 (56, 73)	65 (56, 72)	66 (54, 75)	Z = –1.135	0.257
BMI [M (IQR), kg/m^2^]		24.465 (22.503, 26.649)	24.465 (22.491, 26.573)	24.465 (22.652, 26.667)	Z = –0.234	0.815
LVEF [M (IQR), (%)]		52 (45, 60)	52 (45, 60)	52 (45, 59)	Z = –1.530	0.126
Hemoglobin [M (IQR), g/L]		136 (124, 148)	135 (124, 148)	138 (121, 151)	Z = –0.772	0.440
White Blood Cell count [M (IQR), ×10^9^/L]		7.890 (6.140, 9.942)	7.840 (6.110, 9.740)	8.140 (6.340, 10.550)	Z = –2.128	0.033
Neutrophil count [M (IQR), ×10^9^/L]		5.470 (4.000, 7.388)	5.440 (3.990, 7.280)	5.610 (4.080, 7.780)	Z = –1.457	0.145
Lymphocyte count [M (IQR), ×10^9^/L]		1.560 (1.170, 1.940)	1.560 (1.160, 1.920)	1.600 (1.220, 2.040)	Z = –1.279	0.201
Monocyte count [M (IQR), ×10^9^/L]		0.520 (0.380, 0.680)	0.510 (0.380, 0.670)	0.560 (0.410, 0.725)	Z = –3.412	<0.001
Platelet count [M (IQR), ×10^9^/L]		192.000 (155.000, 229.000)	192.000 (156.000, 227.000)	191.000 (153.000, 237.000)	Z = –0.857	0.391
Fasting blood glucose [M (IQR), mmol/L]		6.240 (5.078, 7.220)	6.250 (5.070, 7.400)	6.130 (5.085, 6.960)	Z = –1.029	0.304
Triglycerides [M (IQR), mmol/L]		1.550 (1.120, 2.083)	1.500 (1.100, 2.000)	1.810 (1.265, 2.490)	Z = –5.459	<0.001
Total Cholesterol [M (IQR), mmol/L]		4.320 (3.610, 4.960)	4.300 (3.600, 4.960)	4.354 (3.770, 4.915)	Z = –1.010	0.312
High-density lipoprotein Cholesterol [M (IQR), mmol/L]		1.030 (0.870, 1.200)	1.050 (0.880, 1.220)	0.970 (0.795, 1.125)	Z = –5.341	<0.001
Low-density lipoprotein Cholesterol [M (IQR), mmol/L]		2.690 (2.058, 3.250)	2.680 (2.030, 3.260)	2.700 (2.190, 3.200)	Z = –0.696	0.486
Lipoprotein a [M (IQR), g/L]		190.200 (109.925, 308.200)	188.400 (111.800, 302.400)	193.900 (103.100, 326.900)	Z = –0.077	0.938
Apolipoprotein A1 [M (IQR), g/L]		1.240 (1.100, 1.390)	1.250 (1.110, 1.400)	1.190 (1.030, 1.370)	Z = –4.406	<0.001
Apolipoprotein B [M (IQR), g/L]		0.940 (0.760, 1.110)	0.930 (0.750, 1.100)	0.953 (0.800, 1.140)	Z = –2.222	0.026
Albumin [M (IQR), g/L]		40.909 (37.900, 44.300)	40.909 (38.000, 44.300)	41.300 (37.600, 44.350)	Z = –0.196	0.844
ALT [M (IQR), U/L]		35.000 (23.000, 45.000)	35.000 (23.000, 43.000)	34.000 (22.000, 50.000)	Z = –0.845	0.398
AST [M (IQR), U/L]		65.000 (28.000, 108.250)	66.000 (28.000, 106.000)	58.000 (29.000, 112.000)	Z = –0.023	0.982
Creatinine [M (IQR), μmol/L]		77.250 (65.150, 90.000)	75.000 (63.000, 88.000)	94.000 (79.700, 116.000)	Z = –14.446	<0.001
C-reactive protein [M (IQR), mg/L‌]		17.319 (5.582, 17.319)	17.319 (4.850, 17.319)	17.319 (10.995, 17.319)	Z = –3.153	0.002

M, median; BMI, body mass index; LVEF, left ventricular ejection fraction; ALT, alanine aminotransferase; AST, aspartate aminotransferase.

### 3.2 Comparison of Novel Biomarker Profiles in the Modeling Group

Comparison of novel biomarkers between two groups revealed that the study group showed higher values of PHR, MHR, CHR, IBI, TyG, RC, AIP, NHHR, and non-HDL-C compared to the control group, while LMR and the CALLY index were significantly lower in the study group, as shown in Table 2.

**Table 2.** **Comparison of novel biomarkers between the two groups**.

Variables	Total (n = 1812)	Control group (n = 1489)	Study group (n = 323)	Statistic	*p*
PLR, M (IQR)	118.889 (92.894, 164.934)	119.277 (93.074, 164.901)	117.799 (90.711, 164.878)	Z = –0.520	0.603
NLR, M (IQR)	3.465 (2.390, 5.178)	3.459 (2.416, 5.188)	3.540 (2.320, 5.162)	Z = –0.156	0.876
LMR, M (IQR)	3.038 (2.195, 4.100)	3.062 (2.240, 4.167)	2.896 (2.084, 3.901)	Z = –2.142	0.032
PAR, M (IQR)	4.680 (3.830, 5.669)	4.674 (3.846, 5.623)	4.722 (3.733, 5.918)	Z = –1.000	0.317
PHR, M (IQR)	183.892 (138.824, 240.780)	182.353 (137.278, 231.915)	196.522 (145.658, 266.422)	Z = –3.858	<0.001
MHR, M (IQR)	0.505 (0.346, 0.710)	0.491 (0.337, 0.689)	0.571 (0.401, 0.822)	Z = –5.186	<0.001
CHR, M (IQR)	15.192 (5.836, 19.680)	14.922 (4.558, 19.243)	16.652 (10.796, 22.203)	Z = –4.874	<0.001
SII, M (IQR)	653.528 (426.989, 1025.207)	655.056 (427.088, 1010.351)	633.857 (417.034, 1066.055)	Z = –0.399	0.690
SIRI, M (IQR)	1.775 (1.044, 3.129)	1.741 (1.035, 2.973)	1.971 (1.090, 3.493)	Z = –1.891	0.059
AISI, M (IQR)	338.289 (183.052, 623.929)	334.122 (182.582, 599.741)	355.712 (190.175, 767.349)	Z = –1.925	0.054
CALLY, M (IQR)	4.287 (2.861, 11.989)	4.380 (2.904, 13.235)	3.982 (2.553, 7.530)	Z = –2.238	0.025
IBI, M (IQR)	48.282 (19.586, 80.942)	48.282 (17.702, 78.232)	48.262 (28.410, 99.304)	Z = –2.353	0.019
TyG, M (IQR)	3.888 (3.712, 4.050)	3.879 (3.702, 4.037)	3.930 (3.772, 4.087)	Z = –3.533	<0.001
TyG-BMI, M (IQR)	95.479 (86.853, 104.360)	95.208 (86.018, 104.312)	96.016 (89.502, 104.627)	Z = –1.878	0.060
RC, M (IQR)	0.460 (0.300, 0.660)	0.450 (0.290, 0.630)	0.530 (0.340, 0.750)	Z = –4.838	<0.001
AIP, M (IQR)	0.504 (0.396, 0.579)	0.502 (0.393, 0.574)	0.517 (0.409, 0.595)	Z = –2.006	0.045
NHHR, M (IQR)	3.091 (2.288, 3.934)	3.086 (2.264, 3.861)	3.214 (2.366, 4.229)	Z = –2.833	0.005
Non-HDL-C, M (IQR)	3.190 (2.490, 3.790)	3.180 (2.470, 3.750)	3.290 (2.565, 3.935)	Z = –2.007	0.045
PNI, M (IQR)	49.217 (45.100, 53.050)	49.150 (45.100, 53.050)	49.550 (44.955, 52.750)	Z = –0.092	0.926

PLR, platelet-to-lymphocyte ratio; NLR, neutrophil-to-lymphocyte ratio; LMR, lymphocyte-to-monocyte ratio; PAR, platelet-to-albumin ratio; PHR, platelet-to-HDL cholesterol ratio; MHR, monocyte-to-HDL cholesterol ratio; CHR, high-sensitivity C-reactive protein (hs-CRP)-to-HDL cholesterol ratio; SII, systemic immune-inflammation index; SIRI, systemic inflammation response index; AISI, aggregate index of systemic inflammation; CALLY, CRP-albumin-lymphocyte index; IBI, inflammatory burden index; TyG, triglyceride-glucose index; TyG-BMI: TyG index × body mass index; RC, remnant cholesterol; AIP, atherogenic index of plasma; NHHR, non-HDL-C/HDL-C ratio; Non-HDL-C, total cholesterol − HDL-C; PNI, prognostic nutritional index.

### 3.3 LASSO Regression Analysis

Variables showing statistical significance in Tables 1,2 were included in the LASSO regression analysis. These comprised: male sex, history of hypertension, history of alcohol consumption, WBC, MON, TG, HDL-C, ApoA1, ApoB, Cr, CRP, PHR, MHR, CHR, IBI, TyG, RC, AIP, NHHR, non-HDL-C, LMR, and the CALLY index.

**Fig. 2.**
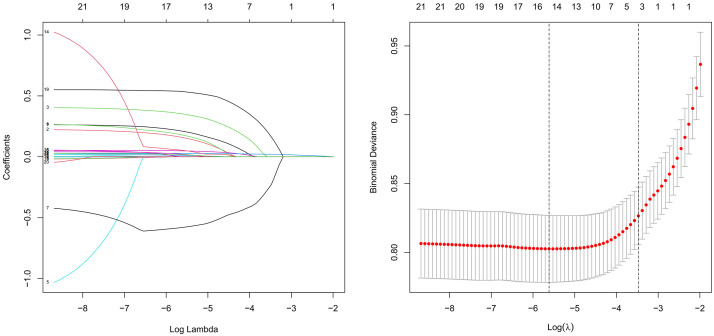
**Least absolute shrinkage and selection operator (LASSO) regression**.

The LASSO regression results indicated that the following variables were associated with HUA in patients with coronary heart disease: male sex, history of hypertension, alcohol consumption, WBC, TG, HDL-C, ApoB, Cr, LMR, PHR, MHR, CALLY index, RC, and NHHR. The variable selection process is illustrated in Fig. 2.

### 3.4 Spearman Analysis

**Fig. 3.**
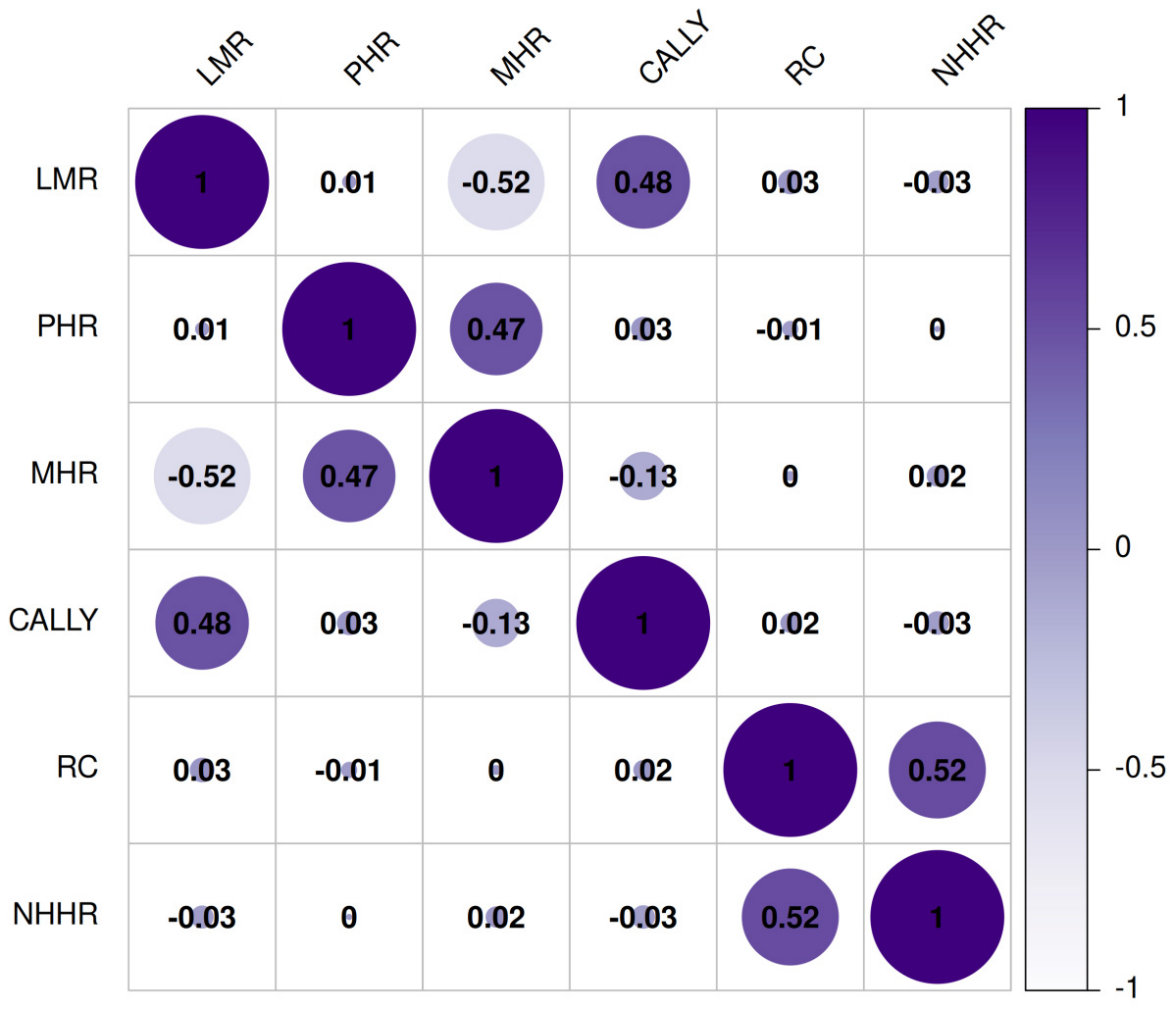
**Spearman analysis of novel biomarkers**.

Spearman analysis was performed to assess relationships among the novel biomarkers and exclude factors with strong multicollinearity. The analysis revealed a moderate correlation between NHHR and RC (*r_s_* = 0.52, *p* < 0.001). The VIF for these two indicators was 1.46, shown in Fig. 3.

### 3.5 Multiple Logistic Regression Analysis

Variables identified as significant in the LASSO regression analysis were included in the multivariate logistic regression model. These variables were: male sex, history of hypertension, history of alcohol consumption, WBC, TG, HDL-C, ApoB, Cr, LMR, PHR, MHR, CALLY index, RC, and NHHR.

The results demonstrated that TG, history of heavy alcohol consumption, Cr, MHR, and RC were independent risk factors for HUA in CHD patients (*p* < 0.05), as detailed in Table 3.

**Table 3.** **Multiple logistic regression analysis of independent risk factors**.

Variables	***b***	SE(*b*)	***z***	*p*	OR (95% CI)
Drinking	0.468	0.145	3.234	0.001	1.596 (1.202, 2.119)
Triglycerides	0.092	0.029	3.194	0.001	1.097 (1.036, 1.160)
Creatinine	0.030	0.002	11.943	<0.001	1.030 (1.025, 1.035)
MHR	0.369	0.160	2.305	0.021	1.447 (1.057, 1.981)
RC	0.595	0.130	4.588	<0.001	1.812 (1.406, 2.337)

*b*, regression coefficient; SE(*b*), standard error; *z*, Z statistic.

### 3.6 Development and Validation of a Visual Prediction Model

**Fig. 4.**
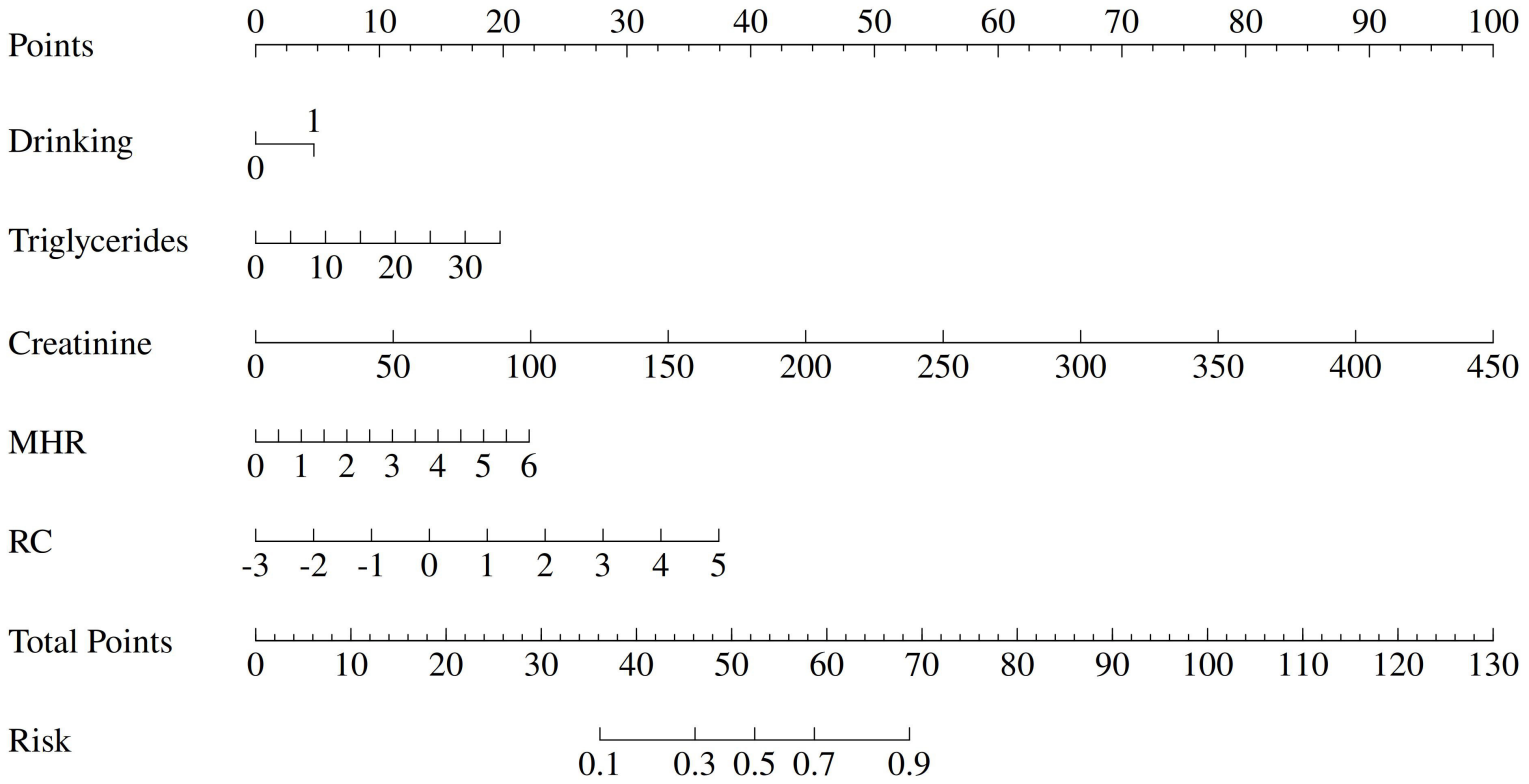
**Nomogram**.

Based on the multivariate logistic regression results, a nomogram was developed to predict the risk of HUA in patients with new-onset CHD. The model incorporated the following independent predictors: history of heavy alcohol consumption (assigned as: yes = 1, no = 0), TG, Cr, MHR, and RC, as shown in Fig. 4.

**Fig. 5.**
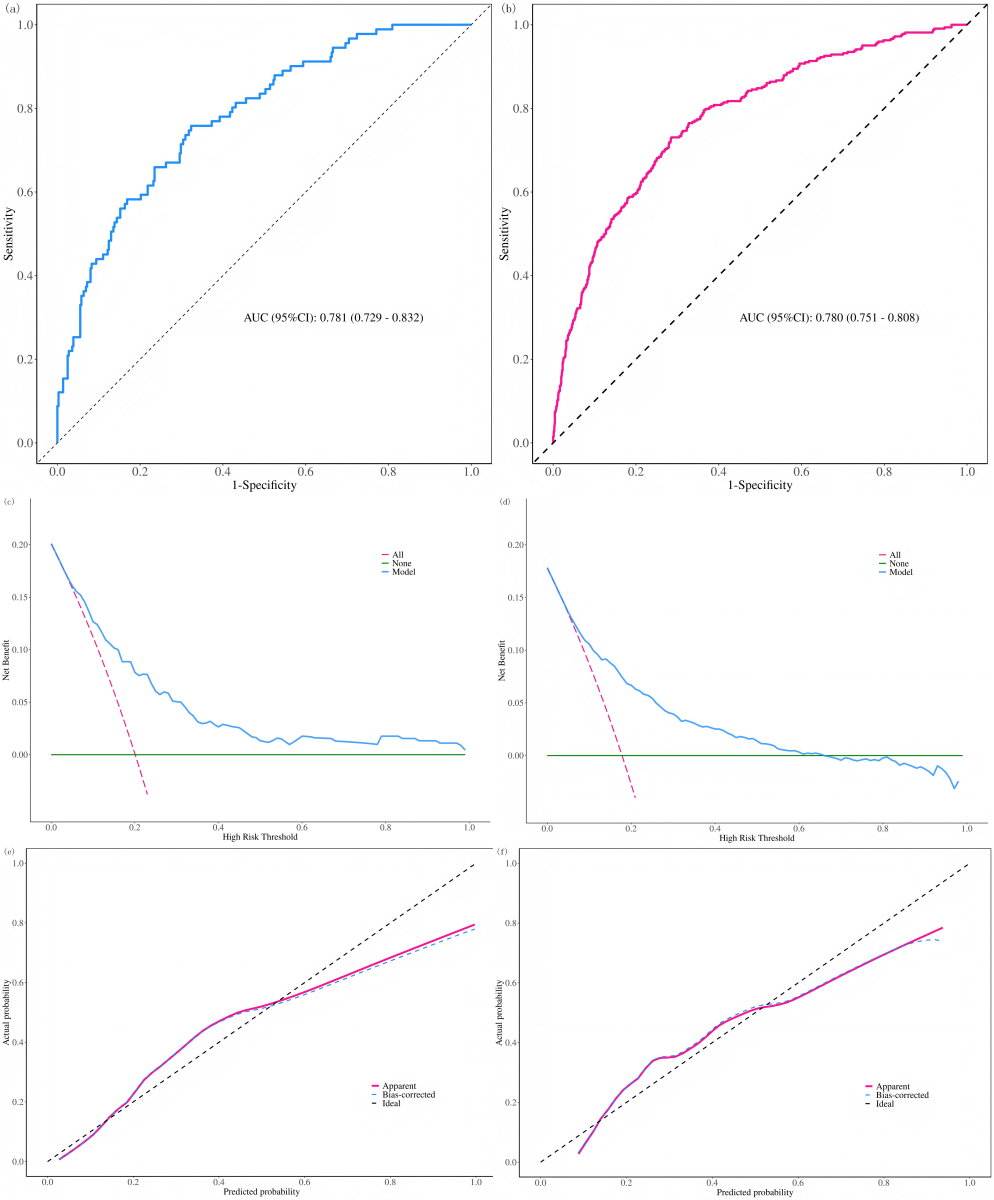
**Evaluation of the predictive model**. (a) Receiver operating characteristic (ROC) curve of modeling cohort. (b) ROC curve of validation cohort. (c) Decision curve analysis (DCA) of modeling cohort. (d) DCA of validation cohort. (e) Calibration curve (Hosmer–Lemeshow test) of modeling cohort. (f) Calibration curve of validation cohort.

The predictive performance of the nomogram was evaluated using receiver operating characteristic (ROC) curve analysis. The AUC was 0.781 (95% CI: 0.729–0.832) in the modeling group and 0.780 (95% CI: 0.751–0.808) in the validation group, indicating good discriminative ability (Fig. 5a,b).

DCA demonstrated that the net benefit was observed when the HUA risk threshold ranged from 0.22 to 1.00 in the modeling group and from 0.20 to 0.66 in the validation group (Fig. 5c,d).

The Hosmer-Lemeshow goodness-of-fit test showed no statistically significant difference between predicted and observed outcomes in either the modeling (*p* = 0.056) or validation (*p* = 0.100) groups. Calibration curves further confirmed good agreement between predicted probabilities and actual observations in both cohorts (Fig. 5e,f), supporting the model’s calibration accuracy.

### 3.7 Subgroup and Restricted Cubic Spline (RCS) Analyses

**Fig. 6.**
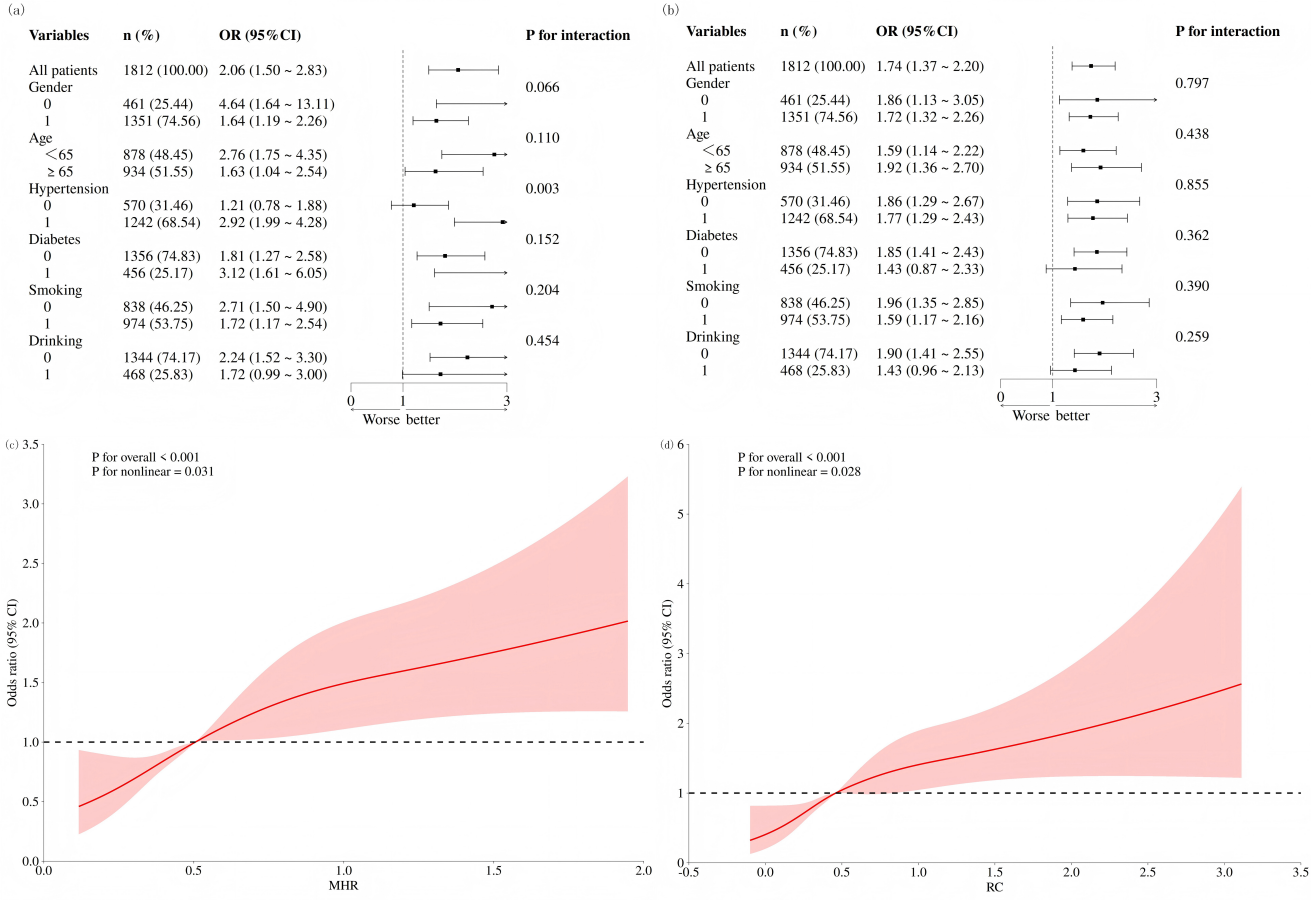
**Subgroup analysis and restricted cubic spline (RCS) regression**. (a) Subgroup analysis of MHR. (b) Subgroup analysis of RC. (c) RCS curve for MHR. (d) RCS curve for RC.

To further investigate the relationship of MHR and RC with the incidence of HUA, subgroup analyses were performed in the modeling cohort (assignment: 1 = yes; 0 = no). The results indicated that when MHR was analyzed as a continuous variable, a significant interaction was observed in the hypertension subgroup. In other subgroups—including sex, age, diabetes, smoking, and alcohol use—MHR consistently showed a significant positive association with HUA incidence. Similarly, RC as a continuous variable exhibited a significant positive correlation with HUA across all subgroups, including sex, age, hypertension, diabetes, smoking, and alcohol use, as shown in Fig. 6a,b.

After adjusting for triglyceride levels, history of heavy alcohol consumption, and creatinine levels, RCS analysis revealed a nonlinear positive association between both MHR and RC and the incidence of HUA, as presented in Fig. 6c,d.

## 4. Discussion

The present study identified MHR (OR = 1.447) and RC (OR = 1.812) as factors associated with HUA in patients with new-onset CHD, among 18 novel biomarkers evaluated. Furthermore, RCS analysis demonstrated a positive dose-response relationship between both MHR and RC and the prevalence of HUA.

HUA is closely linked to heightened cardiovascular risk. As the end product of purine metabolism, UA is involved in various physiological processes. Elevated UA levels can activate inflammatory and oxidative stress responses [6], promoting the release of pro-inflammatory cytokines such as tumor necrosis factor-α (TNF-α) and interleukin-1β (IL-1β) [7]. Furthermore, one study indicated that HUA is associated with an increased 10-year risk of atherosclerotic cardiovascular disease (ASCVD). Specifically, the likelihood of elevated ASCVD risk was 1.3 times higher in men (95% CI: 1.11, 1.52) and 4.34 times higher in women (95% CI: 3.16, 5.91) with HUA compared to those without HUA. UA also serves as an independent predictor of all-cause and cardiovascular mortality in patients with fatal myocardial infarction, heart failure, and cardiometabolic diseases [8].

MHR serves as a comprehensive indicator of systemic inflammation and lipid homeostasis, with an elevated MHR potentially reflecting concurrent enhancement of monocyte activity and impairment of the anti-inflammatory function of HDL. Activated monocytes release pro-inflammatory cytokines, induce vasoconstriction, promote the recruitment of nonspecific immune cells to the vascular wall, and further stimulate the proliferation and activation of other cells within the vessel wall—particularly vascular smooth muscle cells. In contrast, HDL demonstrates a strong capacity to inhibit the expression of adhesion molecules on endothelial surfaces, thereby effectively preventing monocyte recruitment to the arterial wall [9]. Additionally, HDL suppresses monocyte functional activity and interferes with their differentiation into macrophages, consequently limiting the amplification of subsequent inflammatory responses [10]. Once activated, monocytes can differentiate into macrophages and release multiple inflammatory cytokines, thereby exacerbating local inflammation [11,12].

In recent years, RC has garnered significant attention as an emerging lipid metabolism indicator. It refers to the sum of cholesterol in all triglyceride-rich lipoproteins beyond LDL-C and HDL-C, primarily comprising very low-density lipoproteins, intermediate-density lipoproteins, and chylomicron remnants. A prospective study demonstrated [13] that during a median follow-up of 4 years, residual cholesterol levels in CHD patients showed a significant positive correlation with new-onset HUA (OR = 1.23, 95% CI: 1.07, 1.40). Mechanistically, residual cholesterol can cross the endothelial barrier into the vascular wall, interact with the extracellular matrix, and become trapped. Elevated RC levels not only enhance penetration of arterial walls but may also be more readily taken up by smooth muscle cells and macrophages compared to LDL, thereby promoting inflammatory responses [14].

MHR and RC may be linked to HUA through shared metabolic and inflammatory pathways. On one hand, HUA itself can promote oxidative stress and a state of low-grade inflammation, which may influence monocyte activation and HDL function, thereby elevating MHR. On the other hand, increased RC levels reflect dysregulated lipid metabolism. Recent studies suggest that lipid abnormalities may interfere with uric acid excretion by affecting renal hemodynamics or promoting local inflammation [15,16]. Notably, hypertriglyceridemia may indirectly facilitate purine metabolism and uric acid production by enhancing hepatic fatty acid synthesis [16], which could partially explain the positive correlation observed in this study between triglycerides and HUA.

The nomogram constructed in this study incorporated key factors including MHR, RC, triglycerides, creatinine, and alcohol consumption. Multivariate analysis confirmed that triglycerides (OR = 1.097), creatinine (OR = 1.030), MHR (OR = 1.447), RC (OR = 1.812), and alcohol consumption (OR = 1.596) collectively formed a cluster of factors associated with HUA in patients with newly diagnosed CHD. These findings align with several recent studies: alcohol consumption is a recognized risk factor for HUA [17]; dyslipidemia—especially high triglycerides and low HDL-C—is closely linked to the development and progression of HUA [16,18]; and elevated creatinine levels reflect renal functional status. HUA contributes to renal injury by inducing oxidative stress, endothelial dysfunction, and alterations in intrarenal hemodynamics, ultimately leading to nephron loss. Furthermore, the presence of HUA in patients with CKD may indicate significant impairment of uric acid excretion, primarily involving reduced glomerular filtration and enhanced tubular reabsorption [19,20].

There is a recognized link between hypertension and HUA [21]. Subgroup analysis results indicated that the association between MHR and HUA was more pronounced in the hypertensive population (*p* < 0.05). As a common and highly prevalent chronic condition, hypertension significantly increases the risk of cardiovascular and cerebrovascular adverse cvents in patients with CHD [22]. Regarding pathological mechanisms, serum UA can activate the RAS, induce renal vascular damage, and subsequently promote elevated blood pressure. A further study demonstrated [23] a clear dose-response relationship between serum uric acid levels and the relative risk of hypertension, suggesting that UA may exert a continuous promotive effect on the development and progression of hypertension.

## 5. Limitations

First, as a single-center study, the findings may limit their generalizability to other populations. Second, due to the cross-sectional design, the study can only demonstrate associations between HUA and identified risk factors, rather than establish causality. Future research should further validate the association between uric acid levels and cardiovascular risk across different thresholds in multicenter, prospective study designs. Lastly, with the rising prevalence of chronic diseases beyond CHD, future studies should consider more diverse patient populations to better understand the distribution and determinants of HUA.

## 6. Conclusion

In summary, this retrospective analysis of 2265 patients with new-onset CHD identified triglycerides, creatinine, alcohol consumption, MHR, and RC as key associated factors for HUA in this population, with MHR and RC being newly established biomarkers for this association. Based on these indicators, the constructed nomogram demonstrated favorable predictive performance, serving as a reliable clinical instrument for assessing HUA risk in patients with newly diagnosed CHD. Additionally, it offers fresh insights into the inflammatory-metabolic interplay that underlies the co-occurrence of hyperuricemia and cardiovascular disease. Notably, this study employed the conventional hyperuricemia threshold (≥420 μmol/L) [24]. However, recent evidence increasingly indicates that even lower serum uric acid levels (e.g., ≥360 μmol/L) may also correlate with elevated cardiovascular risk, especially among patients with acute coronary syndrome and in the general population. This suggests that the association between uric acid and cardiovascular risk might be continuous rather than strictly threshold-dependent. Future research applying lower cutoff values could further clarify the role of uric acid in subclinical coronary heart disease and its associated comorbidities.

## Data Availability

The datasets used and analysed during the current study are available from the corresponding authors on reasonable request.
